# Neutrophils and Lymphocytes Can Help Distinguish Asymptomatic COVID-19 From Moderate COVID-19

**DOI:** 10.3389/fcimb.2021.654272

**Published:** 2021-10-15

**Authors:** Xuefeng Gu, Ling Sha, Shaofeng Zhang, Duo Shen, Wei Zhao, Yongxiang Yi

**Affiliations:** ^1^ Medical School, Southeast University, Nanjing, China; ^2^ Nanjing Infectious Disease Center, The Second Hospital of Nanjing, The Affiliated Hospital of Nanjing University of Chinese Medicine, Nanjing, China; ^3^ Department of Neurology, Nanjing Drum Tower Hospital, The Affiliated Hospital of Nanjing University Medical School, Nanjing, China

**Keywords:** COVID-19, logistic regression, asymptomatic infection, neutrophils, lymphocytes, DCA

## Abstract

**Introduction:**

Asymptomatic coronavirus disease 2019 (COVID-19) and moderate COVID-19 may be the most common COVID-19 cases. This study was designed to develop a diagnostic model for patients with asymptomatic and moderate COVID-19 based on demographic, clinical, and laboratory variables.

**Methods:**

This retrospective study divided the subjects into 2 groups: asymptomatic COVID-19 (without symptoms, n = 15) and moderate COVID-19 (with symptoms, n = 57). Demographic characteristics, clinical data, routine blood tests, other laboratory tests, and inpatient data were collected and analyzed to compare patients with asymptomatic COVID-19 and moderate COVID-19.

**Results:**

Comparison of the asymptomatic COVID-19 group with the moderate COVID-19 group yielded the following results: the patients were younger (P = 0.045); the cluster of differentiation (CD)8+ (cytotoxic) T cell level was higher (P = 0.017); the C-reactive protein (CRP) level was lower (P = 0.001); the white blood cell (WBC, P < 0.001), neutrophil (NEU, P = 0.036), lymphocyte (LYM, P = 0.009), and eosinophil (EOS, P = 0.036) counts were higher; and the serum iron level (P = 0.049) was higher in the asymptomatic COVID-19 group. The multivariate analysis showed that the NEU count (odds ratio [OR] = 2.007, 95% confidence interval (CI): 1.162 - 3.715, P = 0.014) and LYM count (OR = 9.380, 95% CI: 2.382 - 36.934, P = 0.001) were independent factors for the presence of clinical symptoms after COVID-19 infection. The NEU count and LYM count were diagnostic predictors of asymptomatic COVID-19. This diagnostic prediction model showed high discriminatory power, consistency, and net clinical benefits.

**Conclusions:**

The proposed model can distinguish asymptomatic COVID-19 from moderate COVID-19, thereby helping clinicians identify and distinguish patients with potential asymptomatic COVID-19 from those with moderate COVID-19.

## Introduction

The coronavirus disease 2019 (COVID-19) outbreak began in early December 2019 in Wuhan, China (Zhu et al., 2020). On January 30, the World Health Organization (WHO) issued a public health emergency of international concern (PHEIC), calling on experts from around the world to work together to control the rapid spread of COVID-19. On February 11, 2020, the WHO officially named the disease caused by severe acute respiratory syndrome coronavirus 2 (SARS-CoV-2) as COVID-19 and then officially declared the COVID-19 outbreak a pandemic on March 11, 2020 (WHO, 2020). As of September 12, 2020, more than 18 million individuals have been infected worldwide, with more than 900,000 deaths reported (Chinese Center For Disease Control and Prevention, 2020).

Asymptomatic transmission of COVID-19 has been an important topic. A growing body of evidence shows that many individuals with COVID-19 present no symptoms or only mild symptoms but can spread SARS-CoV-2 (Rivett et al., 2020; Gao et al., 2021). These individuals may have been missed during screening in the early stage of the pandemic due to the limited testing capacity and the nature of asymptomatic infection. However, asymptomatic individuals are currently being identified due to enhanced monitoring and contact screening (Lin et al., 2020). Many respiratory infectious diseases can be asymptomatic. Studies have shown that for human rhinovirus infection, asymptomatic patients outnumber symptomatic patients by a factor of 4 (Granados et al., 2015). For influenza viruses, serological tests indicate that asymptomatic carriers account for 5.2% to 35.5% of all cases (Furuya-Kanamori et al., 2016). Previous studies show that asymptomatic cases account for approximately 9.8% of all cases of Middle East respiratory syndrome coronavirus (MERS-CoV) (Al-Tawfiq and Gautret, 2019). For severe acute respiratory syndrome (SARS), asymptomatic cases (serology) accounted for approximately 13% of all cases (Wilder-Smith et al., 2005). For COVID-19, Kim et al. (2020) showed that 19.2% of 213 patients with COVID-19 were asymptomatic from exposure to admission. In a follow-up study in children under 15 years old in Wuhan, China, 27 of 171 children (15.8%) diagnosed with SARS-CoV-2 infection did not present any symptoms or imaging features of pneumonia (Lu et al., 2020). A simulation study estimated that 17.9% of all infected patients may be truly asymptomatic (Mizumoto et al., 2020).

Asymptomatic individuals may spread the disease, thereby posing a major challenge to infection control. In a prospective study, the infection rate among close contacts of asymptomatic COVID-19 individuals was 4.11%, which is similar to the rate for close contacts of individuals with confirmed COVID-19 (Chen Y. et al., 2020). Therefore, the spread of COVID-19 by asymptomatic individuals must be addressed. Preventing and controlling asymptomatic infection is a major challenge worldwide. Most cases of COVID-19 are moderate, especially outside Wuhan, China, and only a small percentage of patients have severe or critical cases (Lian et al., 2020; Zhang et al., 2020). Accumulating evidence shows that asymptomatic infection is common among COVID-19 patients. Moderate and asymptomatic cases may be the most important COVID-19 cases in the future. Thus, this study was designed to identify (independent) factors of asymptomatic COVID-19 versus moderate COVID-19. The characteristics of patients with moderate and asymptomatic COVID-19 were analyzed, and the results showed that age, cluster of differentiation (CD)8+ (cytotoxic) T cells, C-reactive protein (CRP), white blood cell (WBC) count, neutrophil (NEU) count, lymphocyte (LYM) count, eosinophil (EOS) count, and serum iron were important factors that distinguished patients with asymptomatic COVID-19 from those with moderate COVID-19. A stepwise forward regression analysis showed that NEU and LYM counts were independent predictive factors. This study aimed to establish a low-cost, low-complexity model based on clinical and laboratory variables to help clinicians distinguish patients with asymptomatic COVID-19 from those with moderate COVID-19.

## Materials and Methods

Cases of COVID-19 between January 21, 2020, and February 16, 2020, in the Second Hospital of Nanjing were retrospectively analyzed. A total of 72 patients with SARS-CoV-2 infection were included in the study, 57 of whom had moderate COVID-19 with symptoms (moderate COVID-19 group), while 15 were asymptomatic (asymptomatic COVID-19 group). All patients with COVID-19 were older than 6 years and have no history of agranulocytosis and lymphocytosis. Asymptomatic infection was defined as the absence of any signs or clinical symptoms of COVID-19 despite a positive reverse transcription-polymerase chain reaction (RT-PCR) test result. Many asymptomatic patients with SARS-CoV-2 infection often developed symptoms within a few days and were regarded as having presymptomatic infection (instead of being truly asymptomatic) in this study. SARS-CoV-2 transmission can occur during the incubation period. Therefore, this study was designed to identify factors in patients with truly asymptomatic COVID-19 (asymptomatic throughout the disease course) versus patients with moderate COVID-19, excluding those with presymptomatic COVID-19. Patients with moderate COVID-19 were diagnosed in accordance with *The Guidelines for the Diagnosis and Treatment of COVID-19* (Version 3-5) from the National Health Commission of the People’s Republic of China (China National Health Commission, 2020a; China National Health Commission, 2020b; China National Health Commission, 2020c); the criteria included fever, respiratory symptoms, and imaging signs of pneumonia. All patients in the 2 groups had complete clinical data and a positive RT-PCR test result at admission (baseline). A nasopharyngeal swab was used to collect samples from each patient with suspected COVID-19. RNA collection and extraction were conducted following standard procedures, and the results were interpreted according to the instructions provided with the nucleic acid test (NAT) kit for SARS-CoV-2. The SARS-CoV-2 open reading frame (ORF1ab) and the nucleocapsid protein (N)-encoding gene were used to design the primers and probe targets. The RT-PCR cycle threshold (Ct) is the cycle number at which the fluorescence generated within a reaction crosses the fluorescence threshold for a positive test; a low Ct indicates a relatively high viral load. The diagnostic criteria were based on the recommendations from the National Institute for Viral Disease Control and Prevention (NIVDC, China) (National Institute for Viral Disease Control and Prevention, 2020).

Upon admission, a detailed history of exposure was collected, including a history of residence in or travel to Wuhan. Moreover, electronic medical records were searched to retrieve relevant information, including demographics, clinical characteristics, history of comorbidities, smoking, history of chronic drinking, symptoms, laboratory tests, chest computed tomography (CT), and Ct values over the disease course. At admission, a nasopharyngeal swab was used for COVID-19 testing. Pharyngeal swab specimens were collected on admission day and every other day thereafter for the COVID-19 virus test. All patients in the 2 groups were hospitalized and isolated. They received antiviral therapy including lopinavir/ritonavir, darunavir/cobicistat, or Arbidol combined with interferon-alpha (IFN-α). The discharge criteria were as follows: normal temperature for 3 days or more, significant improvement in respiratory symptoms, significant absorption of pulmonary inflammation (per imaging), and 2 consecutive negative NAT results for SARS-CoV-2 (at least 1 day apart between 2 tests). All samples were processed in the clinical laboratory of the Second Hospital of Nanjing. Two physicians (X.F.G. and W.Z.) independently reviewed the data. This study was approved by the Ethics Committee of The Second Hospital of Nanjing and complied with the principles outlined in The Declaration of Helsinki. The informed consent process was waived because this study posed no risk or adverse effects on subject rights.

### Statistical Analysis

SPSS 19.0 (SPSS, Inc., Chicago IL, USA) and R software (v3.6.0) were used to process and analyze the data. Categorical variables are expressed as N (%), and the chi-square test and Fisher’s exact test (for limited data) were used for comparisons between the 2 groups. Normally distributed continuous data are expressed as the mean ± standard deviation (SD) and were analyzed with the independent samples t-test or paired samples t-test (for paired samples). Nonnormally distributed continuous data are expressed as the medians (interquartile range [IQR]) and were analyzed with the Mann-Whitney test or Wilcoxon signed-rank test (for paired samples). Variables with nonsignificant intergroup differences, variables with too few terminal events to calculate the odds ratio (OR), and variables that had collinearity with NEU count and LYM count were excluded from the multivariate analysis. Stepwise forward regression analysis was performed to identify major diagnostic factors for asymptomatic COVID-19 versus moderate COVID-19. The likelihood ratio, determined by the maximum partial likelihood estimate, was used as the basis for excluding variables. The “boot” package was used for bootstrap resampling of the predictive model. The clinical data of 72 patients were searched to build a new internal validation dataset, a recognized method for internal validation of a predictive model. Internal validation was estimated using the bootstrap method with 1,000 repetitions. Bootstrap is a powerful, computer-based method for statistical to form a sampling distribution just from only one sample data. This method is recommended for internal validation of prognostic models (Austin and Tu, 2012; Gu et al., 2020; Chen et al., 2021; Guerrini et al., 2021). An ROC curve is a graph showing the performance of a classification model at all classification thresholds that illustrates the diagnostic ability of a binary classifier system. The “pROC” R package was used to plot the Non-parametric receiver operating characteristic curve (ROC), and the area under the ROC curve (AUC) was used to evaluate the diagnostic accuracy of the predictive model. there may be some problems in the estimation of the binormal parameter; for example, the ROCKIT program does not converge, and the estimated value of parameter b may be infinite. When the sample size is small and the test results are not well distributed among the possible response categories, some grids often have 0 values. Therefore, the parameter method algorithm may not converge, and the required ROC curve index cannot be obtained. Various approaches have been applied to address this deficiency of the binormal model, including several non-binormal and nonparametric approaches that do not exhibit this deficiency (DeLong et al., 1988; Wieand et al., 1989). Non-parametric methods do not have any distribution assumptions and are an ideal alternative for ROC curve analysis. In this study, in the R software we used, in pROC, ROC curves and associated areas under the ROC curve (AuROCs) were established using the non-parametric, trapezoidal approximation method (Fawcett, 2006). The Hosmer-Lemeshow goodness of fit test was used to calculate the consistency between the predicted probability and the observed probability. A calibration curve was plotted to assess the calibration of the diagnostic prediction model. Then, decision curve analysis (DCA) was used to evaluate the model from the perspective of clinical applicability. All statistical analyses were two-sided, and P < 0.05 was considered statistically significant.

## Results

### Demographic Information and Clinical Characteristics

**Table 1** shows that patients with asymptomatic COVID-19 were younger than those with moderate COVID-19, suggesting that asymptomatic infection is more likely to occur in younger populations (P = 0.045). No significant intergroup differences were observed for sex, history of illness (hypertension, diabetes, and heart disease), smoking, history of chronic drinking, and history of residence in or travel to Wuhan, China. Notably, no significant difference in viral load (aggregated Ct value of the ORF1ab region in the FAM channel) at admission (baseline) was observed between patients with asymptomatic COVID-19 and those with moderate COVID-19 (P = 0.656), suggesting that the presence of clinical symptoms may be related to host immune response rather than viral load.

**Table 1:** Baseline demographic, clinical, and laboratory characteristics of patients with asymptomatic COVID-19 and patients with moderate COVID-19.

Variables	Asymptomatic COVID-19 (n = 15)	Moderate COVID-19 (n = 57)	P value
**Demographics and clinical characteristics**			
Age (years), mean ± SD	36.33 ± 19.54	46.05 ± 15.55	0.045*
Male, n (%)	5 (33.3)	29 (50.88)	0.226
Hypertension, n (%)	1 (6.67)	7 (12.28)	1.000
Diabetes mellitus, n (%)	1 (6.67)	3 (5.26)	1.000
Heart disease, n (%)	1 (6.67)	0 (0.00)	0.208
Smoking, n (%)	2 (13.33)	5 (8.77)	0.630
History of chronic drinking, n (%)	0 (0.00)	4 (7.02)	0.573
History of residence in or travel to Wuhan, n (%)	5 (33.33)	25 (43.86)	0.462
**Laboratory tests**			
FAM channel (Ct value), median (IQR)	27.00 (25.00-34.00)	31.00 (24.50-34.00)	0.656
CD3+ T cells (cell/μl), mean ± SD	1375.73 ± 672.84	1031.65 ± 386.13	0.075
CD4+ T cells (cell/μl), mean ± SD	697.93 ± 387.96	570.11 ± 230.97	0.239
CD8+ T cells (cell/μl), median (IQR)	479.00 (315.00-699.00)	319.00 (222.50-520.00)	0.017*
Th/Ts (CD4+/CD8+), mean ± SD	1.36 ± 0.67	1.71 ± 0.85	0.140
CD45+ lymphocyte count (cell/μl), mean ± SD	1972.67 ± 876.62	1492.84 ± 527.54	0.059
ESR (mm/h), median (IQR)	7.00 (5.00-9.00)	10.00 (5.50-25.00)	0.162
PCT (ng/ml), median (IQR)	0.017 (0.012-0.078)	0.020 (0.014-0.054)	0.546
CRP (mg/L), median (IQR)	0.87 (0.29-2.96)	7.90 (2.02-21.90)	0.001*
WBC (10^9^/L), median (IQR)	5.63 (5.04-6.61)	4.32 (3.72-4.82)	<0.001*
NEU (10^9^/L), median (IQR)	3.17 (2.45-4.08)	2.53 (2.08-2.90)	0.036*
LYM (10^9^/L), mean ± SD	1.82 ± 0.69	1.27 ± 0.44	0.009*
EOS (10^9^/L), median (IQR)	0.05 (0.01-0.15)	0.02 (0.00-0.05)	0.036*
BAS (10^9^/L), median (IQR)	0.01 (0.01-0.02)	0.005 (0.01-0.02)	0.032*
MONO (10^9^/L), mean ± SD	0.42 ± 0.16	0.37 ± 0.12	0.148
HGB (g/L), mean ± SD	132.13 ± 16.92	135.11 ± 16.03	0.530
PLT (10^9^/L), mean ± SD	224.33 ± 68.36	180.93 ± 53.99	0.011*
TBIL (μmol/L), median (IQR)	10.60 (7.60-13.40)	11.90 (9.15-15.35)	0.450
ALT (U/L), median (IQR)	14.70 (11.30-25.70)	20.40 (14.45-30.00)	0.197
AST (IU/L), median (IQR)	20.60 (16.50-21.70)	21.80 (18.45-29.60)	0.140
M-AST isoenzyme (U/L), median (IQR)	2.40 (2.20-2.90)	2.50 (1.60-3.25)	0.803
CHE (U/L), mean ± SD	8121.47 ± 1727.04	7435.54 ± 1784.68	0.187
LDH (IU/L), median (IQR)	191.00 (175.00-256.00)	213.00 (184.50-265.50)	0.247
ALP (IU/L), median (IQR)	67.30 (57.50-84.10)	67.10 (55.75-80.55)	0.895
TBA (μmol/L), median (IQR)	3.60 (2.70-5.60)	5.30 (2.85-7.35)	0.289
RBP (mg/L), median (IQR)	21.00 (18.70-31.90)	20.30 (16.40-24.85)	0.172
ALB (g/L), mean ± SD	45.38 ± 2.39	44.23 ± 3.54	0.240
UA (μmol/L), mean ± SD	220.07 ± 85.88	231.63 ± 72.63	0.599
LA (mmol/L), median (IQR)	2.86 (2.50-3.21)	2.95 (2.61-3.21)	0.632
GLU (mmol/L), median (IQR)	4.59 (4.20-5.05)	4.61 (4.19-5.15)	0.682
BUN (mmol/L), mean ± SD	3.46 ± 1.05	3.33 ± 0.87	0.615
CRE (μmol/L), median (IQR)	47.00 (41.00-79.00)	61.00 (48.00-70.00)	0.282
Serum potassium (mmol/L), mean ± SD	3.99 ± 0.50	3.82 ± 0.40	0.161
Serum Sodium (mmol/L), median (IQR)	140.20 (139.20-141.10)	139.10 (136.80-141.00)	0.092
Serum calcium (mmol/L), mean ± SD	2.23 ± 0.13	2.17 ± 0.13	0.101
Serum magnesium (mmol/L), mean ± SD	0.93 ± 0.12	0.92 ± 0.13	0.847
Serum iron (μmol/L), median (IQR)	14.70 (10.60-22.70)	10.90 (7.65-15.90)	0.049*
MAO (U/L), mean ± SD	4.11 ± 1.25	4.47 ± 1.57	0.413
AFU (U/L), mean ± SD	24.40 ± 6.52	23.00 ± 5.80	0.420
ADA (U/L), median (IQR)	6.00 (5.00-9.00)	7.00 (6.00-9.00)	0.186
CPK (U/L), median (IQR)	56.00 (41.00-75.00)	57.00 (41.00-97.00)	0.835
IMA (U/mL), median (IQR)	67.00 (64.00-76.00)	72.00 (66.50-78.50)	0.163
FIB (g/L), median (IQR)	2.42 (2.12-2.92)	2.97 (2.38-3.63)	0.084
APTT (second), median (IQR)	27.70 (25.40-32.30)	31.10 (28.80-35.15)	0.079

*P < 0.05. P values were calculated by an independent samples t-test, the Mann-Whitney U test, the χ^2^ test, or Fisher’s exact test, as appropriate. ADA, adenosine deaminase; AFU, α-L-fucosidase; ALB, albumin; ALP, alkaline phosphatase; ALT, alanine aminotransferase; APTT, activated partial prothrombin time; AST, aspartate aminotransferase; BAS, basophils; BUN, blood urea nitrogen; CHE, cholinesterase; COVID-19, coronavirus disease 2019; CPK, creatine phosphokinase; CRE, creatinine; CRP, C-reactive protein; Ct, cycle threshold; EOS, eosinophil; ESR, erythrocyte sedimentation rate; FAM, open reading frame 1ab (ORF1ab) region; FIB, fibrinogen; GLU, glucose; HGB, hemoglobin; IMA, ischemia-modified albumin; IQR, interquartile range; LA, lactic acid; LDH, lactic dehydrogenase; LYM, lymphocyte; MAO, monoamine oxidase; MONO, monocyte; NEU, neutrophil; PCT, procalcitonin; PLT, platelet; RBP, retinol binding protein; SD, standard deviation; TBA, total bile acid; TBIL, total bilirubin; Th/Ts, T helper/T suppressor cell ratio; UA, uric acid; WBC, white blood cell.

### Laboratory Test Results

**Figure 1:**
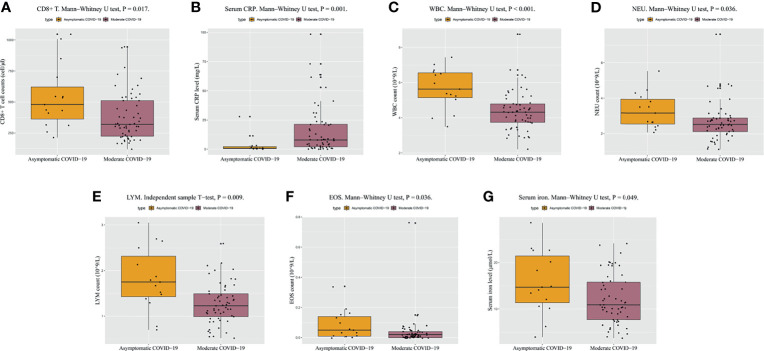
Clinically relevant biochemical results at admission for patients with asymptomatic COVID-19 and moderate COVID-19. **(A–G)** Differences in CD8+ T cell counts, CRP, WBC counts, NEU counts, LYM counts, EOS counts, and serum iron between patients with asymptomatic COVID-19 and patients with moderate COVID-19. P < 0.05 is considered significant. COVID-19, coronavirus disease 2019; CRP, C-reactive protein; EOS, eosinophil; LYM, lymphocyte; NEU, neutrophil; WBC, white blood cell.

Regarding the absolute counts of peripheral blood T lymphocyte subsets, the median level of CD8+ T cells was in the normal range in both groups but was lower than the normal value (238/µL) in 28.07% (16/57) of the patients in the moderate COVID-19 group and in only 2 patients in the asymptomatic COVID-19 group. The level of CD8+ T cells in the moderate COVID-19 group was significantly lower than that in the asymptomatic COVID-19 group (P = 0.017) (**Table 1** and **Figure 1A**), suggesting that a low level of CD8+ T cells was common in patients with moderate COVID-19, whereas most patients with asymptomatic COVID-19 had normal CD8+ T cell immunity. No significant intergroup differences were observed for other immune indicators such as CD3+ T cells, CD4+ T cells, CD45+ lymphocyte count, and the T helper/T suppressor cell ratio (Th/Ts). Thus, the CD8+ T cell level influences cellular immune responses and may be important in the presence of clinical symptoms in individuals with SARS-CoV-2 infection.

Compared with the asymptomatic COVID-19 group, the moderate COVID-19 group had significantly lower WBC counts (P < 0.001, **Table 1** and **Figure 1B**), absolute NEU counts (P = 0.036, **Table 1** and **Figure 1C**), absolute LYM counts (P = 0.009, **Table 1** and **Figure 1D**), EOS counts (P = 0.036, **Table 1** and **Figure 1E**), basophil counts (P = 0.032, **Table 1**), and platelet (PLT) counts (P = 0.011, **Table 1**) and significantly higher CRP levels (P = 0.001, **Table 1** and **Figure 1F**). The percentages of patients with a low WBC count, NEU count, and LYM count were 38.60% (22/57), 22.81% (13/57), and 14.04% (8/57) in the moderate COVID-19 group, respectively, and only 13.33% (2/15), 0.00% (0/15), and 13.33% (2/15) in the asymptomatic COVID-19 group, respectively. The percentages of patients with high CRP were 71.93% (41/57) in the moderate COVID-19 group and only 20.00% (3/15) in the asymptomatic COVID-19 group. No significant intergroup differences were observed in the erythrocyte sedimentation rate, procalcitonin, monocyte count, and hemoglobin. These hematological parameter results indicated that for patients with asymptomatic COVID-19, the numbers of different blood cell types were higher, and the CRP level was more stable.

Serum iron was the only blood biochemistry parameter (among 20+ parameters) with a significant intergroup difference between patients with asymptomatic COVID-19 and those with moderate COVID-19 (P = 0.049, **Table 1** and **Figure 1G**). The mean or median values for the other parameters in both groups were in the normal ranges, with no significant intergroup differences.

### Construction of a Diagnostic Model That Distinguishes Patients With Asymptomatic COVID-19 From Those With Moderate COVID-19

A multivariate analysis was performed to build a diagnostic model based on potentially significant factors identified in the univariate analysis. Among all the patients, the PLT count was abnormal (high or low) in only 2 patients, and the basophil count was normal in all patients. Therefore, these 2 variables were excluded from the multivariate analysis because any intergroup differences (if any) within the normal range may not be clinically relevant. The -log2 likelihood ratio was used to select variables, and a stepwise forward regression analysis was performed; 2 independent factors, NEU count (OR = 2.007, 95% confidence interval (CI): 1.162 - 3.715, P = 0.014) and LYM count (OR = 9.380, 95% CI: 2.382 - 36.934, P = 0.001), were identified for the final diagnostic model (**Table 2**). The OR values indicate that the probability of asymptomatic infection increases by 2.077-fold or 9.380-fold with each unit increase in the NEU count or LYM count, respectively.

**Table 2:** Multivariate logistic regression analysis of patients with asymptomatic COVID-19 and patients with moderate COVID-19.

Variables	B	SE	OR	95%CI	P value
NEU	0.731	0.297	2.077	1.162-3.715	0.014*
LYM	2.239	0.699	9.380	2.382-36.934	0.001*

*P < 0.05. B, regression coefficient; CI, confidence interval; OR, odds ratio; SE, standard error.

### Evaluation of the Accuracy of the Predictive Diagnostic Model Combining NEU and LYM Counts

**Figure 2:**
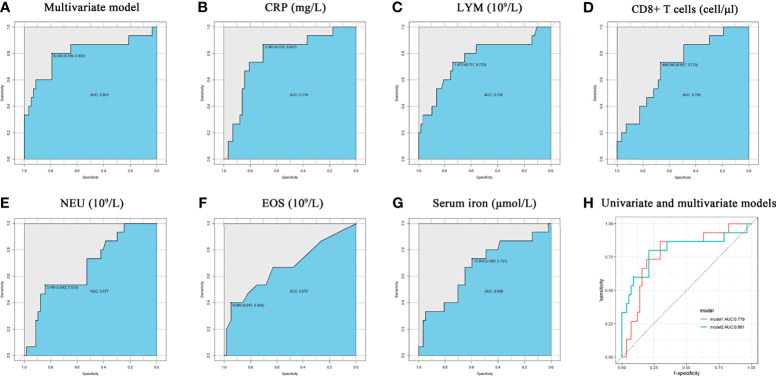
Non-parametric ROC analysis of meaningful variables. **(A–G)** the model combining NEU and LYM counts, CRP, LYM counts, CD8+ T cell counts, NEU counts, EOS counts, and serum iron. all P < 0.05. **(H)** Comparison between ROC curves for CRP univariate model and multivariate model combining NEU and LYM counts. Model1 represents the CRP univariate model, and model2 represents the multivariate model combining NEU and LYM counts. ROC, Receiver operating curves.

Non-parametric ROC curve analysis was performed on the constructed predictive diagnostic model, and the results showed that the area under the curve (AUC) of the model combining NEU and LYM counts was 0.801 (95% CI: 0.650 - 0.953, P < 0.001; **Figure 2A**). Following a reported method (Pastor et al., 2019). Non-parametric ROC analysis was performed on meaningful variables, and the results showed that CRP had the best classification performance for distinguishing SARS-CoV-2 infection with or without clinical symptoms (AUC = 0.779, 95% CI: 0.649-0.909; P = 0.001; **Figure 2B**). Among the other analyzed variables, LYM count had an AUC of 0.756 (95% CI: 0.604 - 0.907, P = 0.002; **Figure 2C**), and CD8+ T cells had an AUC of 0.702 (95% CI: 0.560 - 0.843, P = 0.017; **Figure 2D**), showing good discriminatory performance. The AUCs of NEU count, EOS count, and serum iron were 0.677 (95% CI: 0.528 - 0.825, P = 0.036; **Figure 2E**), 0.675 (95% CI: 0.506 - 0.843, P = 0.038; **Figure 2F**), and 0.666 (95% CI: 0.503 - 0.829, P = 0.049; **Figure 2G**), respectively; these 3 variables had poor accuracy in identifying SARS-CoV-2 infection with or without clinical symptoms. The AUC for age was 0.646 (95% CI: 0.470 - 0.821, P = 0.084), indicating no statistically significant difference and poor predictive accuracy.

CRP was the single indicator that could best distinguish COVID-19 patients with or without clinical symptoms. The univariate CRP model had a Youden’s index of 3.365 mg/L for diagnosis prediction, with a sensitivity of 70.2% and a specificity of 86.7%. In contrast, the multivariate model combining NEU and LYM counts had a cutoff score of 0.254 for Youden’s index, a sensitivity of 78.9%, and a specificity of 80.0%. Both models could accurately distinguish SARS-CoV-2 infection with or without clinical symptoms, but the model combining NEU and LYM counts had higher sensitivity and discriminative power. Compared with the other univariate models, the model combining NEU and LYM counts had a greater AUC and higher sensitivity and specificity (**Figure 2H**). Therefore, the model combining NEU and LYM counts is feasible and has higher application value.

### Validation of the Predictive Diagnostic Model Distinguishing Patients With Asymptomatic COVID-19 From Those With Moderate COVID-19

**Figure 3:**
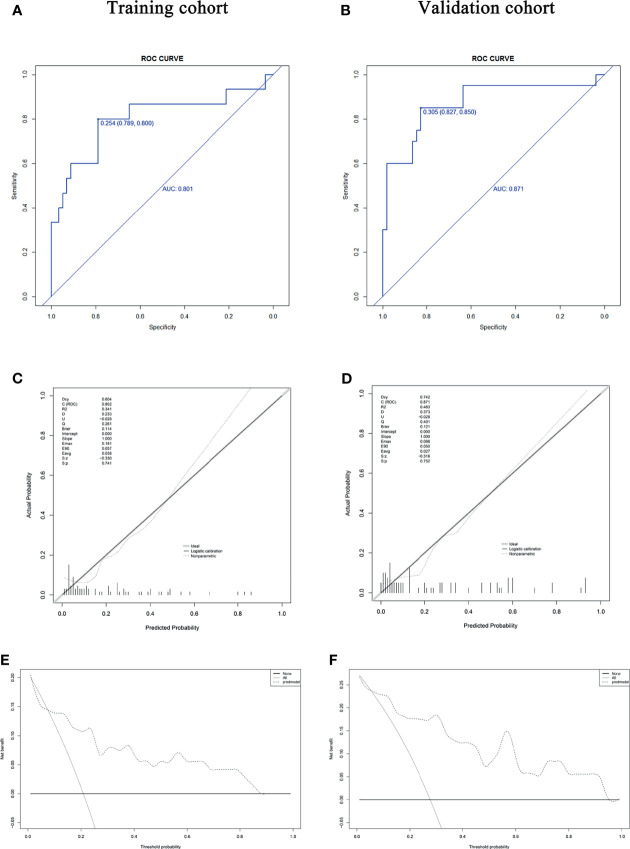
The diagnostic value, concordance, and clinical relevance of the diagnostic model. **(A, B)** The AUC indicates the discriminatory performance of the diagnostic model in the training and validation cohorts. **(C, D)** Calibration test of the diagnostic model in the training and validation cohorts. The fitting curve by regression analysis (black solid line) approximates the standard curve (gray line), with no significant difference (P = 0.741 and P = 0.752, respectively). **(E, F)** DCA of the training and validation cohorts. DCA showed more benefit from using the diagnostic prediction model if the threshold probability was in the ranges of ≤ 2% and 8-87% in the training cohort and a higher overall net benefit if the threshold probability was in the ranges of ≤ 1% and 6-94% in the internal validation cohort. AUC, area under the curve; DCA, decision curve analysis.

The Non-parametric ROC analysis showed that the areas under the curve (AUCs) were 0.801 in the training cohort (**Figure 3A**) and 0.871 in the internal validation cohort (**Figure 3B**) for the asymptomatic COVID-19 group, confirming the good performance of the diagnostic model for asymptomatic COVID-19. **Figures 3C, D** show the calibration curves for the training cohort and the internal validation cohort, respectively. The calibration curves showed good consistency for the diagnostic model, and the Hosmer-Lemeshow test confirmed good calibration (training cohort: χ^2^ = 12.321, P = 0.196; validation cohort: χ^2^ = 15.852, P = 0.070). DCA results showed that the diagnostic prediction model has good clinical application value (**Figures 3E, F**). Therefore, this model can be used to distinguish patients with asymptomatic COVID-19 patients from those with moderate COVID-19.

### Comparison of the Characteristics of Inpatients With Asymptomatic COVID-19 and Moderate COVID-19

During hospitalization, the 15 asymptomatic patients showed overall mild changes on chest CT. Specifically, 2 asymptomatic patients presented normal CT images from admission to discharge, 4 (26.67%) presented streaky opacities in the lungs, and 9 presented ground-glass opacities or patchy high-attenuation patterns in the lungs. When comparing the moderate COVID-19 group with the asymptomatic COVID-19 group (P = 0.173, **Table 3**), no significant intergroup difference in suspected contact time was observed. No significant intergroup differences were observed in the time from a positive NAT result to the first negative NAT result (P = 0.525, **Table 3**) and the time from admission to the last positive NAT result (P = 0.578, **Table 3**), suggesting that the viral load (Ct) at admission (baseline), the time to the first negative NAT result, and the time to final virus clearance were not significantly different between the 2 groups. Overall, these data indicated that dynamic changes in viral load may be unrelated to the presence of clinical symptoms of COVID-19. In this study, no significant intergroup difference was observed in hospital stay (P = 0.703, **Table 3**), but a significant intergroup difference was observed in healthcare expenses (P = 0.013, **Table 3**) because patients with moderate COVID-19 had more clinical symptoms and required more diagnostic tests and treatments.

**Table 3:** Characteristics of inpatients with asymptomatic COVID-19 and inpatients with moderate COVID-19.

Variables	Asymptomatic COVID-19 (n = 15)	Moderate COVID-19 (n = 57)	P value
Suspected contact time (days)	15 (9 -17)	10 (8 -15.5)	0.173
Time from the positive NAT result to the first negative NAT result (days)	6.00 (3.00-10.00)	7.00 (4.00-10.00)	0.525
Time from admission to the last positive NAT result (days)	11.00 (6.00-20.00)	11.00 (4.00-17.00)	0.578
Hospital stay (days)	22.00 (13.00-28.00)	20.00 (12.00-27.50)	0.703
Inpatient costs (CNY)	17239.10 (11426.35-22269.27)	30189.60 (16350.83-42955.95)	0.013*

*P < 0.05. CNY, Chinese Yuan; NAT, nucleic acid test.

### Changes in the Levels of Important Blood Cell Types of Asymptomatic COVID-19 and Moderate COVID-19 Patients From Admission to Discharge

**Figure 4:**
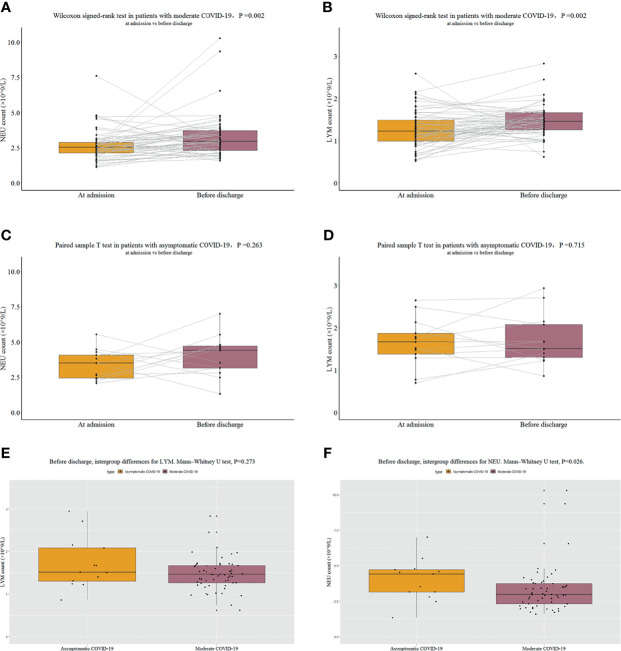
NEU count and LYM count. **(A)** NEU counts at admission and before discharge in patients with moderate COVID-19. **(B)** LYM counts at admission and before discharge in patients with moderate COVID-19. **(C)** NEU counts at admission and before discharge in patients with asymptomatic COVID-19. **(D)** LYM counts at admission and before discharge in patients with asymptomatic COVID-19. **(E)** Comparison of LYM counts before discharge in patients with moderate COVID-19 and patients with asymptomatic COVID-19. **(F)** Comparison of NEU counts before discharge in patients with moderate COVID-19 and patients with asymptomatic COVID-19.

After administration of active antiviral and symptomatic treatments, the laboratory tests were repeated for all patients (except 2 asymptomatic patients due to their short hospital stay) before discharge. The results showed that the NEU count (P = 0.002, **Figure 4A**) and LYM count (P = 0.002, **Figure 4B**) significantly increased after active treatment in the moderate COVID-19 group, whereas no significant changes were observed from admission to discharge in the asymptomatic COVID-19 group [NEU count (P = 0.263, **Figure 4C**) and LYM count (P = 0.715, **Figure 4D**)]. Clinicians are still debating the treatment of asymptomatic infections. This study showed that for patients with asymptomatic COVID-19, antiviral therapy may not have appreciable effects on the functions of important blood cells because asymptomatic patients often have a normal NEU count and LYM count at admission (baseline); therefore, asymptomatic patients may not require active antiviral treatment. At admission, significant intergroup differences were observed in both the NEU count and LYM count, whereas at discharge, no significant intergroup difference was observed in the LYM count (P = 0.273, **Figure 4E**); meanwhile, a significant intergroup difference was still observed for the NEU count (P = 0.026, **Figure 4F**). These results suggest that for patients with moderate COVID-19, the LYM count may return to normal more quickly after treatment, whereas the NEU count may require longer to recover.

## Discussion

COVID-19 is an acute infectious disease caused by SARS-CoV-2. Most patients experience clinical symptoms, but some patients have no symptoms and are known as asymptomatic patients. Distinguishing patients with asymptomatic COVID-19 from those with moderate COVID-19 is important. In this study, a simple model with 2 variables, NEU count and LYM count, was established to guide the diagnosis of asymptomatic COVID-19 and moderate COVID-19, which showed good diagnostic performance, consistency, and net clinical benefits.

Many studies have shown that asymptomatic patients are younger (He et al., 2020; Ma et al., 2020). In this study, patients with asymptomatic COVID-19 were younger than patients with moderate COVID-19, indicating that age may be an important factor associated with COVID-19 severity, which may be related to the immune response and other underlying pathologies in patients of different ages. For example, COVID-19 is usually milder in children, resulting in mild or no clinical symptoms (Lu et al., 2020), due to their better adaptive immune response (Grimsholm et al., 2020) and lower concentration of angiotensin-converting enzyme 2 (ACE-2) receptors (Wang A. et al., 2020). More importantly, this study showed no significant difference in viral load at admission between patients with asymptomatic COVID-19 and those with moderate COVID-19, which is consistent with the findings of Zou et al. (2020). Moreover, no significant intergroup differences were observed in the time from a positive NAT result (on day 1) to the first appearance of 2 consecutive negative NAT results and the time from admission to the last positive NAT result. These data indicate that viral load may be unrelated to clinical symptoms of COVID-19.

An intact immune system is the basis for proper immune function. T lymphocyte subsets in blood are the most important component of the immune system. Studies have shown that (Sungnak et al., 2020) viral entry-associated genes and genes involved in innate immunity are coexpressed in nasal epithelial cells. Therefore, if the early immune response is sufficient to inhibit viral replication and further prevents virus from entering the lungs, the infected individual may have only mild or even no symptoms. An effective innate response also gives the body sufficient time to initiate an effective T cell response, which helps reduce the severity of symptoms (mild or no symptoms) in patients. A high level of CD8+ T cells in peripheral blood provides clues to a sustained and effective T-cell-mediated immune response (Maggi et al., 2020). This study showed that most asymptomatic patients (except 2 patients with a low CD8+ T cell level) had a normal CD8+ T cell level in their peripheral blood, whereas the percentage of patients with moderate COVID-19 with a low CD8+ T cell level was significantly higher (28.07%). Moreover, other than CD8+ T cells, no significant intergroup differences were observed in other T lymphocyte subsets, such as CD3+ T cells, CD4+ T cells, and CD45+ T cells. Urra et al. (2020) showed that a low CD8+ T cell level, but not CD4+ T cell level, was related to the severity and clinical progression of COVID-19. Yin et al. (2021) retrospectively analyzed the clinical data of 48 COVID-19 subjects and found that CD8+ T cells in severe or critical case patients were lower than those in asymptomatic and mild symptom patients. Mallajosyula et al. (2021) reported that CD8+ T cells are much more abundant in COVID-19 patients with mild disease than in those with a more severe illness. These findings support and are consistent with our conclusion. These data indicate that CD8+ T cell level is the most important immune factor that distinguishes patients with asymptomatic COVID-19 from those with moderate COVID-19.

Han et al. (2020) studied serum cytokines in COVID-19 patients and found that the CRP level was significantly higher in COVID-19 patients than in healthy individuals. Chen W. et al. (2020) found that the serum CRP level was positively correlated with COVID-19 severity. In a study of 298 patients, CRP was also identified as an independent predictor of adverse prognosis in patients with COVID-19 (Luo et al., 2020). These studies indicate that CRP is an important indicator of the disease condition of COVID-19 patients. This study showed that the CRP level in patients with moderate COVID-19 was significantly higher than that in patients with asymptomatic COVID-19 (the difference was significant), suggesting that CRP may be used to predict the presence of clinical symptoms of COVID-19.

Blood leukocytes are an important part of the body’s defense system, and infection status can be predicted by measuring WBC levels. Guan et al. (2020) surveyed 1,099 patients and found that 83.2% of the patients with COVID-19 had a decreased lymphocyte count at the time of hospital admission. In addition, 36.2% of the COVID-19 patients had a decreased blood platelet level, and 33.7% of the COVID-19 patients had a decreased leukocyte count. Sun et al. (2020) conducted a study with 116 patients with COVID-19. The leukocyte count, lymphocyte count, eosinophil count, platelet count, and hemoglobin level were all found to be decreased in COVID-19 patients. In addition, neutropenia can predict a poor prognosis for COVID-19 patients (Wang D. et al., 2020) and is also an independent predictive factor for a poor prognosis in COVID-19 patients (Luo et al., 2020). Lymphopenia is a factor related to poor prognosis in disease development (Ruan et al., 2020; Yang et al., 2020). The reasons have been analyzed; in terms of neutrophils, one theory posits that the primary cause of the exaggerated host response in patients with severe COVID-19 lies in the abnormal activation of neutrophils. Neutrophil infiltration into the pulmonary capillaries and neutrophil extravasation into the alveolar space have been observed in autopsy samples (Barnes et al., 2020). Neutrophils are the main cells of the innate immune system. When lung infection occurs, neutrophils rapidly chemotactically aggregate to the sites of infection, where they kill the pathogens through oxidative bursts and phagocytosis (Schönrich and Raftery, 2016). Another mechanism of action involves the formation of neutrophil extracellular traps (NETs) (Fuchs et al., 2007). NETs may cause organ damage and death in patients with COVID-19. Currently, the role of NETs in COVID-19 is a potentially important area worthy of exploration (Mozzini and Girelli, 2020). The neutrophils in peripheral blood may decline mainly because of the redistribution of neutrophils or the destruction of neutrophils by antibodies in circulation. Xu Z. et al. (2020) conducted a study on the lymphocytes in COVID-19 patients. The pathological anatomy results for deceased individuals also showed significant inflammatory infiltration of monocytes (predominantly lymphocytes) in the lung interstitium. The significant decrease in the number of lymphocytes in COVID-19 patients may also be related to the redistribution and increased depletion of lymphocytes (Yao et al., 2020). In addition, in the acute phase of virus-induced lung infection, EOSs accumulate in the infected tissues to resist viral infection, resulting in a low EOS count in peripheral blood (Samarasinghe et al., 2014). This may also apply to patients with COVID-19. Moreover, virus may directly inhibit the proliferation of nucleated cells in bone marrow, resulting in significantly low LYM, granulocyte and PLT counts in peripheral blood. He et al. (2020) analyzed the clinical characteristics of 12 patients with asymptomatic COVID-19 and found that the LYM count was low in 2 patients and normal in other patients and that the WBC count was normal in all patients. Xu T. et al. (2020) conducted a study of 15 asymptomatic patients and found that at admission, the WBC count was normal in all patients and the LYM count was low in only 1 patient, consistent with the results of this study. Among the 15 asymptomatic patients in this study, at admission, the WBC count was low in only 2 patients, the LYM count was low in only 2 patients, and the NEU count was normal in all patients.

Previous investigations indicate that the age and gender effect on the lymphocytes and neutrophils count in normal and infectious individuals. Some researchers (Arumanayagam et al., 1987) think that in males, WBC counts show a statistically significant increase with age. In females, however, all blood cell indices remain relatively unchanged up to 70 years of age. Other studies (Omuse et al., 2018) have drawn different conclusions. Except for the decline in monocyte levels with increasing age among female participants, all other CBC parameters did not change with age and sex. Oloume et al. (2019) found that the median white blood cell count was 4.1 × 10^9^/L in men and 4.6 × 10^9^/L in women (p = 0.008) in a healthy adult population in Yaoundé, Cameroon. Karazawa and Jamra (1989) showed that women have higher white blood cell counts than men in healthy people in Brazil. In contrast, Arumanayagam et al. (1987) found that the mean WBC value in males was significantly higher than that in females in healthy Hong Kong Chinese adults. In addition, some studies have shown that sex is unrelated to white blood cells. Koram et al. (2007) reported that the white blood cell (WBC) count did not significantly differ between males and females in the Akuapem North district in healthy adults in the Eastern Region of Ghana. We believe that the difference in these results may be due to social, environmental, and nutritional factors, age, sex, race, occupation, and other individual characteristics.

In our study, the young individuals in the asymptomatic group (mean ± SD, 36.33 ± 19.54 years) had higher WBC counts, while the older individuals in the moderate group (mean ± SD, 46.05 ± 15.55 years) had lower WBC counts. Previous studies have shown that WBC counts statistically significantly increase with age (Giorno et al., 1980; Arumanayagam et al., 1987). The conclusion of our research contradicts the conclusions of previous studies involving healthy people. We found that this finding may be due to a decline in the WBC count (which should have increased) due to the disease severity. Many reports suggest that neutrophils and lymphocytes are related to the disease severity or prognosis of COVID-19 patients (Sun et al., 2020; Yang et al., 2020; Guan et al., 2020; Ruan et al., 2020; Wang D. et al., 2020). In this study, our results show that the percentage of female individuals in the asymptomatic COVID-19 group was 66.66% compared to 49.12% in the moderate COVID-19 group. There was a higher percentage of women in the asymptomatic group. We used a chi-square test to compare the sex baseline levels, and the results showed that there was no significant difference in sex (P = 0.226, P > 0.05). The balance of the baseline data was comparable. We think that although the sex ratios in the two groups differed, there is no significant difference in sex between the two groups, and they are comparable.

In short, WBC count and WBC differential counts may be abnormal in COVID-19 patients and may be important factors of COVID-19 severity. This study showed that compared with patients with asymptomatic COVID-19, the total WBC count, NEU count, LYM count and EOS count were significantly lower in patients with moderate COVID-19. The multivariate analysis showed that the NEU count and LYM count were independent factors in distinguishing patients with asymptomatic COVID-19 from those with moderate COVID-19, with an important diagnostic value.

In addition, this study showed that serum iron in patients with moderate COVID-19 was significantly decreased (10.90 µmol/L [IQR 7.65-15.90]) and was lower than that in patients with asymptomatic COVID-19 (P = 0.049), suggesting that serum iron was another factor in distinguishing patients with asymptomatic COVID-19 from those with moderate COVID-19. Previous studies have shown that serum iron in SARS patients is significantly lower than that in healthy individuals (Song et al., 2004). This is because interleukin (IL)-6 produced during acute and chronic inflammation upregulates hepcidin, a negative regulator of iron absorption, thereby resulting in low serum iron (Baron et al., 2020).

In summary, predicting asymptomatic COVID-19 cases and distinguishing them from moderate COVID-19 cases are very important; asymptomatic and moderate COVID-19 cases are the 2 most common types of COVID-19 and the main infection sources during the COVID-19 pandemic. Some researchers believe that asymptomatic patients can spread the disease to the same extent as COVID-19 patients with symptoms (Yin and Jin, 2020). However, most asymptomatic patients often do not seek medical attention due to an absence of obvious clinical symptoms or lack of awareness regarding prevention. Therefore, epidemiological methods, such as screening of close contacts, epidemiological surveys of COVID-19 clusters and follow-up surveys of infection sources, are important for identifying asymptomatic patients, who are the largest hidden source in the resurgence of COVID-19 and pose a major challenge to infection control. This study can help facilitate an understanding of the laboratory diagnostic characteristics of patients with asymptomatic COVID-19 to distinguish them from patients with moderate COVID-19. This study identified NEU and LYM counts as independent indicators for distinguishing asymptomatic COVID-19 from moderate COVID-19, which can provide important guidance. This study has some limitations. First, this was a retrospective analysis with potential for recall bias. Second, this was a single-center study; thus, the results are not applicable to the general population due to inherent selection bias of inpatients. Last, the sample size was small, which may affect the interpretation of the study findings. Nevertheless, after including all eligible asymptomatic patients from a designated COVID-19 hospital in Nanjing, we believe that the study population is the most representative population in Nanjing.

## Conclusions

In conclusion, the clinical characteristics of patients with asymptomatic COVID-19 outside Wuhan, China, were described, and a multivariate model to distinguish asymptomatic patients from patients with moderate COVID-19 was established, which may provide new indicators for monitoring COVID-19-infected populations.

## Data Availability Statement

The raw data supporting the conclusions of this article will be made available by the authors, without undue reservation.

## Ethics Statement

This study was approved by the Ethics Committee of the Second Hospital of Nanjing (NO. 2020-LS-ky003). Written informed consent from the participants’ legal guardian/next of kin was not required to participate in this study in accordance with the national legislation and the institutional requirements.

## Authors Contributions

WZ and YY designed the study. XG and SZ collected data. XG and LS performed the data analysis and prepared the figures and tables. XG, LS, and DS completed the statistical analysis. XG drafted the manuscript. and WZ and YY supervised the study. All authors read and approved the final manuscript.

## Conflict of Interest

The authors declare that the research was conducted in the absence of any commercial or financial relationships that could be construed as a potential conflict of interest.

## Publisher’s Note

All claims expressed in this article are solely those of the authors and do not necessarily represent those of their affiliated organizations, or those of the publisher, the editors and the reviewers. Any product that may be evaluated in this article, or claim that may be made by its manufacturer, is not guaranteed or endorsed by the publisher.

## Abbreviations

ACE-2, angiotensin-converting enzyme 2; AUC, area under the curve; CI, confidence interval; COVID-19, coronavirus disease 2019; CRP, C-reactive protein; Ct, cycle threshold; EOS, eosinophil; IQR, interquartile range; LYM, lymphocyte; MERS-CoV, Middle East respiratory syndrome coronavirus; NAT, nucleic acid test; NETs, neutrophil extracellular traps; NEU, neutrophil; NIVDC, National Institute for Viral Disease Control and Prevention; OR, odds ratio; PHEIC, public health emergency of international concern; ROC, receiver operating characteristic; RT-PCR, reverse transcription-polymerase chain reaction; SARS, severe acute respiratory syndrome; SARS-CoV-2, severe acute respiratory syndrome coronavirus 2; SD, standard deviation; Th/Ts, T helper/T suppressor cell ratio; WBC, white blood cell; WHO, World Health Organization.
